# Tumor Resident Memory T Cells: New Players in Immune Surveillance and Therapy

**DOI:** 10.3389/fimmu.2018.02076

**Published:** 2018-09-11

**Authors:** Nina Dumauthioz, Sara Labiano, Pedro Romero

**Affiliations:** Department of Oncology, Faculty of Biology and Medicine, University of Lausanne, Épalinges, Switzerland

**Keywords:** tissue resident memory, vaccination, mucosal route, CD103, cancer prognosis, immunotherapy

## Abstract

Tissue resident memory T cells (Trm) are a subset of memory T cells mainly described in inflammation and infection settings. Their location in peripheral tissues, such as lungs, gut, or skin, makes them the earliest T cell population to respond upon antigen recognition or under inflammatory conditions. The study of Trm cells in the field of cancer, and particularly in cancer immunotherapy, has recently gained considerable momentum. Different reports have shown that the vaccination route is critical to promote Trm generation in preclinical cancer models. Cancer vaccines administered directly at the mucosa, frequently result in enhanced Trm formation in mucosal cancers compared to vaccinations via intramuscular or subcutaneous routes. Moreover, the intratumoral presence of T cells expressing the integrin CD103 has been reported to strongly correlate with a favorable prognosis for cancer patients. In spite of recent progress, the full spectrum of Trm anti-tumoral functions still needs to be fully established, particularly in cancer patients, in different clinical contexts. In this mini-review we focus on the recent vaccination strategies aimed at generating Trm cells, as well as evidence supporting their association with patient survival in different cancer types. We believe that collectively, this information provides a strong rationale to target Trm for cancer immunotherapy.

## Introduction

Tumor-infiltrating lymphocytes (TILs) frequently remain tolerant or display an exhausted phenotype favored by the tumor microenvironment (1–3). Thus, two of the main challenges of current immunotherapy against cancer are generating specific T cells that may effectively target tumor cells and ensuring the induction of long-term anti-tumor protective immune responses. Therapeutic strategies to promote the development of immunological memory have for the most part focused on circulating memory T cells, such as central memory (Tcm) or effector memory (Tem) but have failed so far to consider resident memory cells (Trm).

Trm cells are a long-lasting population frequently characterized by the expression of CD103, CD69, and CD49a surface markers and by the absence of the lymph node homing receptors CD62L and CCR7. The differentiation toward a residency memory program is known to be regulated by TGFβ and IL-15 cytokines, which promote the expression of the transcription factors Hobbit and Blimp1. The upregulation of these molecules induces the silencing of other transcription factors such as KLF2 and TCF1 and proteins involved in tissue egress such as S1PR1 (4). Trm cells are mainly localized in peripheral lymphoid and non-lymphoid tissues such as lung, skin, gastrointestinal and genitourinary tracts (5). Their permanence in these tissues is mainly mediated by the expression of the integrins CD103 and CD49a that bind E-cadherin and collagen respectively. The homing properties of Trm cells can vary depending on the tissue and the chemokine receptor expression patterns. The presence of CCR5 and CXCR3, for instance, is essential for the recruitment of CD8+ Trm cells to the lungs in cancer and infection (6, 7).

Trm cells have been broadly studied in infection and inflammation settings (8), where they are considered as the first immune population to become activated even in the absence of *in situ* antigen recognition (9). Their role in immune surveillance of cancer remains unclear, although there is an increase in the number of publications associating the presence of Trm cells in tumor areas with a favorable outcome. Of note, the importance of this subset of lymphocytes has been described in anti-tumor immunity of skin and mucosal tumors (10, 11). Hence, the development of immunotherapeutic strategies favoring Trm cell generation and function could be critical not only in controlling tumor growth but also, and foremost, in preventing tumor recurrences.

In this mini-review we will cover two main aspects of Trm cells in cancer: the importance of the vaccination route to promote Trm cells against tumor antigens and the evidence substantiating an association of their occurrence to patient survival.

## Vaccination routes that promote trm cells in cancer

The precise procedures for the generation *ad libitum* of tissue resident memory remain still unknown. A recent study found the existence of a common clonal origin for central and resident memory T cells upon immunizing the skin of mice and humans with different components (protein antigen, hapten and non-replicative virus) (12).

Crosspriming dendritic cells seem to be critical for the generation of resident memory in the tissues. In a preclinical infection model, the priming strength and the durability of antigen presentation were reported to play an important role in Trm generation, in an IL-12, IL-15, and CD24 dependent manner (13). Moreover, the absence of crosspriming dendritic cells results in a preferential reduction of Trm cells over circulating memory T cells in both infection and cancer settings (13, 14).

The imprinting of homing properties of tissue resident T cells is also dependent on the priming event. It has been shown that dendritic cells (DCs) can induce different arrays of chemokine receptors and adhesion molecules, such as integrins or selectin-ligands, in CD8 T cells depending on the sites where they uptake the antigens (15). In a glioblastoma preclinical model, the injection of tumor cells by distinct routes (intraperitoneal, intracranial and subcutaneous) was shown to promote different patterns of integrins on specific T lymphocytes isolated from the respective tumor-draining lymph nodes (15). These results suggest a mechanistic explanation of the impact of the immunization route in the generation of Trm cells (16–23).

Despite the fact that many cancers develop at mucosal sites such as the lungs, oral and genitovaginal cavities or the gastrointestinal tube, most of the preclinical cancer vaccines have been administered subcutaneously or intramuscularly, thus without specific targeting of mucosal sites. This may be relevant in advanced diseases with multiple visceral and cutaneous metastases. Indeed, subcutaneous immunization with DCs pulsed with a human melanoma peptide was found to be sufficient to reject subcutaneous melanomas but not lung metastases. However, systemic intravenous immunization prevented lung metastasis in a mechanism involving CD8 T cells that were retained in the spleen (24).

Recent studies carried out by different groups have assessed vaccination at mucosal sites. By using an orthotopic head and neck tumor model expressing the E7 antigen from HPV (TC-1 cell line), it was demonstrated that intranasal vaccination prevents the tumor growth in the oral cavity and in the lungs. Such an effect was not observed with vaccination by the intramuscular route (25). This anti-tumor outcome was mainly dependent on the presence of E7-tetramer positive CD8 T cells expressing mucosal integrins (CD49a and CD103) that were not only found in tumors but also in mediastinal and cervical lymph nodes (25). In a model of cervicovaginal cancer, the generation of CD8 T cells with a resident phenotype was promoted upon intravaginal viral vector-based vaccination, which also boosted circulating tumor-specific T cells. T cells expressing CD103 and CD69 were shown to produce high levels of IFNγ and TNFα at the tumor site (16, 26). In line with this, the combination of intravaginal HPV-based vaccination administered upon intramuscular E7-expressing DNA vaccine, enhanced tumor-specific CD8 T cells in the mucosa of an HPV-cervicovaginal cancer model. The α4β7 integrin expressed by CD8 T lymphocytes was found to be the main integrin responsible for the migration of these cells to the genital mucosa. Clear evidence shows that DCs present at the mucosal site induce the upregulation of α4β7 integrin on CD8 T cells, favoring homing to the tumor (27). By injecting the same tumor cell line in the bladder, another group showed promising anti-tumor effects in a therapeutic intravaginal vaccine. Although in this model the subcutaneous route showed a better outcome, it was proven that the tumor growth control of intravaginal route was due to presence of tumor-specific CD8 T cells in the bladder mucosa. The fact that these CD8 T cells are detectable at later time points upon vaccination indicates that intravaginal vaccination can give rise to the generation of resident memory T cells (28).

However, intravaginal vaccination was not able to prevent growth of tumors in a transplantable vaginal cancer generated by intravaginal instillation of TC-1 cells expressing the E7 antigen (29). Control of the tumor growth was achieved with subcutaneous and intranasal routes in detriment of intravaginal vaccine administration. The clear discrepancy between these results and the previously mentioned related to the intravaginal vaccination route in cervicovaginal cancer might be explained by the type of vaccines employed in each case. In this study mice were injected with a vaccine based on the E7-peptide combined with different adjuvants, while previous studies focused on viral based-vector vaccines containing E7 antigen. Furthermore, the targeting of different subsets of DCs should be taken into account. Given the fact that crosspriming dendritic cells are necessary for the generation of Trm cells, a vaccine that potentially targets these specific DCs may induce more Trm and thus, a better outcome in cancer patients.

In fact, the idea of targeting DCs to promote the induction of CD103 on primed CD8 T cells was further explored in a humanized breast cancer model. In this study, DCs that were reprogrammed via dectin-1 favored the generation of a resident phenotype on CD8 T cells in a TGFβ-dependent manner (30). It was also shown that TLR agonists such as poly(I:C; TLR3) or CpG-ODN (TLR9) induced systemic but not resident memory T cells. In contrast, in a genital tumor model, the intravaginal injection of TLR agonists in combination with an E7 peptide-based vaccine, was reported to promote the recruitment of E7-specific T cells into the tumor. Although the increased recruitment of T cells was proposed to be responsible for tumor regression, the generation of Trm cells by this vaccine was not clearly addressed in this study (31).

Even though Trm cells are broadly present in the skin, their generation in this tissue has not been deeply studied in a skin cancer setting. Up to now, two recent reports describe how a prophylactic vaccination through skin scarification enhanced Trm generation in skin, preventing subcutaneous, and intradermal-injected tumor growth (13, 32).

## CD103+ expression on TILS is associated with patient survival

The integrin αE(CD103)β7 selective for E-cadherin identifies tumor antigen-reactive TILs with more potent effector functions than the CD103 negative TIL subset. Indeed, CD103+ TILs displayed enhanced killing capacity (33) and CD103+ T cells infiltrating glioma had an improved ability to produce granzyme B (34). Upon binding to E-cadherin, CD103+ T cells undergo cytotoxic granule polarization, and degranulation concomitantly to TCR engagement (33, 35). Moreover, the expression of CD103 has been associated with patient survival in diverse cancer types: melanoma (36), non-small cell lung carcinoma (NSCLC) (37, 38), bladder cancer (39), endometrial cancer (EC) (40), breast cancer (41), cervical cancer (42) and high-grade serous ovarian cancer (HGSC) (43, 44, 45, 46) (Table 1).

**Table 1:** Association of patient survival with CD103+ expression on tumor infiltrating lymphocytes (TILs) in different cancer types.

**Tumor**	**CD103+ TILs correlate with survival**	**Treatment**	**Trm cells phenotype**	**Cancer grade**	**No. of patients**	**References**
Glioma	N.A.	(–)	CD103+, Granzyme B+	(–)	6–7	(34)
Melanoma	N.A.	Vaccination	CD103+, CD69+, VLA-1+	III/IV	18	(47)
	Yes	+/–αPD-1	CD103+, CD69+, PD-1^high^, Granzyme B+, KLRG1^low^	III	44	(36)
NSCLC	Yes	Surgery	CD103+, CD69+, PD-1^high^	Early stage	101	(37)
	Yes	(–)	CD103+, CD69+, CD49a+	Early stage	36 and 689	(38)
Bladder	Yes	Surgery	CD103+	Ta-T4	302	(39)
Endometrial	Yes	Surgery	CD103+, PD-1+	FIGO I-IV	305	(40)
Cervix	Yes	Surgery and/or radio(chemo)	CD103+	FIGO IA2-IVA	304	(42)
Breast	Yes	Surgery and radiation or chemotherapy	CD103+	FIGO I-III	424	(41)
HGSC	Yes	Surgery and chemotherapy	CD103+, PD-1+	FIGO I-III	210	(46)
	Yes	Surgery and chemotherapy	CD103+	FIGO II-III	135	(43)
	Yes	Surgery and chemotherapy	CD103+, PD-1+	FIGO IIb	186	(44)
	Yes	Surgery and chemotherapy	CD103+	FIGO I-IV	135	(45)
Colorectal	No	Surgery	CD103+	T1-T4	239	(48)

*N.A., not analyzed; (–), not known*.

At the antigen specific T cell level, a recent study from a phase I clinical trial enrolling melanoma patients vaccinated subcutaneously with a melanoma antigen (Melan-A), analyzed the homing receptors characterizing Melan-A-specific CD8 T cells. The presence of circulating melanoma-specific T cells harboring P-Selectin binding and Very late antigen (VLA-1) correlated with improved patient survival. Moreover, VLA-1+ CD8 T cells were strongly enriched in melanoma metastases (lung, skin and brain) and displayed a Trm phenotype expressing the CD103 and CD69 surface markers (47). Another recent report also focused on melanoma patients, naïve of any treatment or undergoing αPD-1 therapy. They demonstrated an improved survival in 50% of patients with a high number of CD103+ TILs cell compared to 20% in those with lower numbers (36).

A correlation between a high CD103+ TIL density and patient survival was also shown in early stage NSCLC patients. CD103 + CD8 TILs from these tumor biopsies displayed Trm phenotype and expressed high levels of the inhibitory receptors PD-1 and TIM-3 (37). Along the same lines, in NSCLC patients, it was revealed that highly infiltrated tumors (TILs^high^) were enriched for Trm cells and the density of CD103+ CD8 TILs was associated with a favorable outcome (38).

In bladder urothelial cell carcinoma, the density of CD103+ TILs correlated with survival and was inversely linked to the tumor volume (39). Similarly, patients diagnosed with endometrial cancer or cervical cancer had improved survival when a high infiltration of CD103+ TILs was detected (40, 42).

In breast cancer, especially in the basal-like tumor subtype, the patients had improved survival when the tumor was enriched with CD103+ CD8 TILs (41).

The association between CD103+ cells and survival was also established by several studies in high-grade serous ovarian cancer (HGSC) (43–46). It was shown that CD103+ TIL infiltration (46) and the presence of CD103+ or CD3+ intraepithelial lymphocytes (43) correlated with better survival in HGSC patients. Moreover, the stratification of patients according to CD103 or CD3 counts in the tumor, highlighted striking differences according to overall survival: the CD3^high^CD103^high^ group had a 5-years survival rate at 90%, the CD3^low^CD103^high^ at 63% and the CD3^low^CD103^low^ at 0%, thus demonstrating the crucial presence of TILs and the potential power of the CD103 marker to predict patient outcome (43).

CD103+ TIL detection is associated with an improved survival in various mucosal tumor models. However, a study performed with colorectal cancer (CRC) patients did not find any difference in survival comparing intraepithelial CD103+ cell density in the whole cohort. In addition, analysis of high CD103+ density in KRAS WT CRC patients, a subgroup of the cohort, defined a group with unfavorable outcome (48).

Taken together, the presence of high levels of CD103+ TILs is associated with improved patient survival in the majority of the cancer types described here, albeit with the possible exception CRC. This prominent exception remains to be confirmed, including the distinction of CRC subtype. Moreover, the possible mechanistic basis for this unique disconnect may offer further insights into the role of Trm cells in different tumor microenvironments. Of note, Tregs may also express CD103 (49), thus calling for a complete subset analysis of TILs. In this regard, novel high content immunohistochemical technologies may help in providing high resolution TIL analyses.

## Trm cells as targets for cancer immunotherapy

CD8+ CD103+ TILs have been shown to express high levels of PD-1 in various cancers: melanoma (36), lung cancer (37, 38), endometrial adenocarcinoma (40) and HGSC (44, 46). CD8+ CD103+ PD-1+ TILs displayed potent cytokine production (IFNγ and TNFα) after PMA/ionomycin stimulation, thus representing an interesting target for immunotherapy (50). Indeed, it was demonstrated that αPD-1 treatment led to CD103+ TIL expansion in the majority of melanoma patients showing improved survival, confirming that the use of checkpoint blockade may effectively boost this T cell population leading to favorable patient outcomes (36).

Interestingly, the expression of other inhibitory receptors varies in CD8+ CD103+ TILs within different cancer types. For instance, in melanoma CD103+ CD69+ TILs were PD-1+ and LAG-3+ but negative for CTLA-4 (36); in NSCLC CD103+ TILs expressed PD-1 and TIM-3 but with negligible CTLA-4 expression (37) and in HGSC CD103+ TILs did not express TIM-3, CTLA-4 or LAG-3 (50). The different expression profiles of inhibitory receptors on CD103+ TILs warrant further detailed characterization that may serve to guide the selection of the specific immune checkpoint blockade administration, such as monotherapies or combinations.

It was demonstrated that human Influenza-specific Trm cells possess strong proliferative potential after CFSE labeling (51). In addition, secondary effector cells derived from CD8+ CD103+ CD69+ Trm cells displayed enhanced polyfunctionality, since IFNγ, TNFα, and granzyme B production was improved compared to effectors differentiated from CD103+ CD69- or CD103-CD69+ subsets (51). Therefore, it would be of great interest to sort, re-expand, and adoptively transfer (ACT) this T cell population in patients. However, when ACT of Trm cells was attempted in a mouse model, it was met with poor success (52). This failure may be explained in part by poor homing capacities of Trm cells. An approach to counteract the homing intrinsic limitations of Trm cells could be the transfer of this population directly at the required mucosal site. On the other hand, the use of ACT of Tcm cells in mice efficiently led to the generation of a Trm cell population after infection or tumor challenge (13). Thus, the potential of ACT with Tcm cells due to their plasticity could be exploited to favor Trm cell differentiation.

## Concluding remarks

The growing interest in CD8+ Trm cells is illustrated in the number of recent studies aimed at understanding the optimal way to promote their formation in preclinical cancer models. According to reports detailed above, the vaccination route is crucial to boost Trm cell formation at the required site. In several models, the intramucosal vaccination route demonstrated an enhanced potential to generate Trm cells, compared to the conventional intramuscular or subcutaneous routes.

It has also been demonstrated that CD8+ Trm cells may be key players in successful cancer immunotherapy, since their presence in tumor areas is frequently associated with better survival in the majority of cancer types. Due to their localization, mostly at mucosal sites, and surface expression of several inhibitory receptors, such as PD-1, CD103+ TILs represent an appealing target for immunotherapy. Nevertheless, it remains unclear whether the adoptive transfer of these cells would succeed due to their poor homing capacities. However, the enhancement of Trm proliferation and function seems to be critical in combating mucosal cancers where Trm are prevalent. Promising results in cancer vaccination indicate that this approach may be the most productive way to target Trm cells in the clinic, and their performance in combination with immune checkpoint blockade, or other immunotherapy modalities, awaits evaluation in clinical trials.

## Author contributions

ND, SL, and PR discussed and wrote the manuscript.

### Conflict of interest statement

The authors declare that the research was conducted in the absence of any commercial or financial relationships that could be construed as a potential conflict of interest.
